# Plum modulates Myoglianin and regulates synaptic function in *D. melanogaster*

**DOI:** 10.1098/rsob.230171

**Published:** 2023-09-13

**Authors:** Virender K. Sahota, Aelfwin Stone, Nathaniel S. Woodling, Jereme G. Spiers, Joern R. Steinert, Linda Partridge, Hrvoje Augustin

**Affiliations:** ^1^ Department of Biological Sciences, Centre for Biomedical Sciences, Royal Holloway University of London, Egham, Surrey TW20 0EX, UK; ^2^ Institute of Healthy Ageing, and GEE, University College London, Darwin Building, Gower Street, London WC1E 6BT, UK; ^3^ Max Planck Institute for Biology of Ageing, Joseph-Stelzmann-Str. 9b, Cologne 50931, Germany; ^4^ Faculty of Medicine & Health Sciences, Queen's Medical Centre, Nottingham NG7 2UH, UK; ^5^ MRC Toxicology Unit, Hodgkin Building, University of Leicester, Lancaster Road, Leicester LE1 9HN, UK

**Keywords:** plum, myoglianin, *Drosophila melanogaster*, neuromuscular junction, synapse

## Abstract

Alterations in the neuromuscular system underlie several neuromuscular diseases and play critical roles in the development of sarcopenia, the age-related loss of muscle mass and function. Mammalian Myostatin (MST) and GDF11, members of the TGF-β superfamily of growth factors, are powerful regulators of muscle size in both model organisms and humans. Myoglianin (MYO), the *Drosophila* homologue of MST and GDF11, is a strong inhibitor of synaptic function and structure at the neuromuscular junction in flies. Here, we identified Plum, a transmembrane cell surface protein, as a modulator of MYO function in the larval neuromuscular system. Reduction of Plum in the larval body-wall muscles abolishes the previously demonstrated positive effect of attenuated MYO signalling on both muscle size and neuromuscular junction structure and function. In addition, downregulation of *Plum* on its own results in decreased synaptic strength and body weight, classifying Plum as a (novel) regulator of neuromuscular function and body (muscle) size. These findings offer new insights into possible regulatory mechanisms behind ageing- and disease-related neuromuscular dysfunctions in humans and identify potential targets for therapeutic interventions.

## Graphical abstract

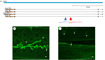

## 1. Introduction

The neuromuscular system is composed of individual motor units, each consisting of a single motor neuron, a neuromuscular junction (NMJ), and muscle fibres innervated by the motor neuron. Diminished motor unit function and decreased muscle volume are hallmarks of several neuromuscular disorders [1] and of sarcopenia, the age-associated loss of skeletal muscle mass and function [2]. Gradual loss of skeletal muscle capacity has been reported in invertebrates, rodents and humans [3–5], with intrinsic mechanisms regulating age-related muscle dysfunction largely conserved across species [6]. Age-related muscle loss is accompanied by progressive modifications in the structure and function of the NMJ, the specialized synapse at the interface between the nervous and muscular system [1], with the resulting uncoupling of the excitation–contraction machinery [7,8]. In mammals, including humans, these modifications include changes in the branching pattern of the motor nerve terminal that contacts the myofibre, fragmented NMJ architecture, impaired synaptic neurotransmitter distribution, and decreased density of presynaptic active zone markers [9–13]. Functionally, aged mammalian NMJs exhibit increased failures in evoked release [14], changes in quantal release [15] and a slowing-down of axoplasmic transport of proteins [16]. Skeletal muscle and NMJ deficits are found in many motoneuronal and neuromuscular disorders, with impaired neurotransmission and muscle wasting characterizing amyotrophic lateral sclerosis (ALS) [17–19], myotonic and muscular dystrophies [20–24] and myasthenia gravis [25,26]. Whether muscle loss precedes or follows the changes in the function of the NMJ is currently unresolved, but animal studies suggest that NMJ remodelling plays a critical role in the progression of sarcopenia [27].

*Drosophila melanogaster* is a convenient and proven model system for studying various aspects of developmental regulation of muscle mass and control of NMJ function [28–30]. *Drosophila* larval glutamatergic NMJs share structural and functional similarities with mammalian junctions [31] and striated muscles in *Drosophila* resemble vertebrate skeletal muscles in structure, function, and protein composition [28]. Previously, we used this model system to investigate the role of *Drosophila* MYO, the muscle- and glia-expressed fly homologue of TGF-β growth factors Myostatin (MST) and GDF11 [32] in regulating synaptic function, muscle size and body weight [33]. MST (also known as GDF-8) is a circulating cytokine that serves as a powerful negative regulator of muscle mass in mammalian species [34,35]. In addition to its MST-like role as an inhibitor of larval weight and muscle size, muscle-derived MYO is a strong negative regulator of neurotransmission, synaptic morphology and the density of critical pre- and post-synaptic components [33].

Plum is a *Drosophila* transmembrane, immunoglobulin superfamily protein [36] and a distant homologue of Protogenin, Sidekick and Nope, mammalian regulators of developmental processes in nervous, muscle and epithelial tissues [37–39]. Protogenin was also associated with attention deficit hyperactivity disorder [40]. Sidekick regulates synaptic connections in the vertebrate retina [41], and Nope is a surface marker for human and murine liver cancers [42,43]. Recently, Plum was identified as a modulator of axon pruning in the *Drosophila* nervous system [36]. Plum genetically interacts and interferes with MYO function, likely by sequestrating MYO [36]. In this study, we examined the interactions between Plum and MYO in regulating larval muscles and NMJ physiology. We identified Plum as a novel modulator of MYO action on NMJ synaptic transmission and muscle size, and an independent regulator of synaptic strength and larval weight.

## 2. Results

### 2.1. Muscle-derived plum regulates NMJ synapse strength independently and by modulating MYO

**Figure 1.**
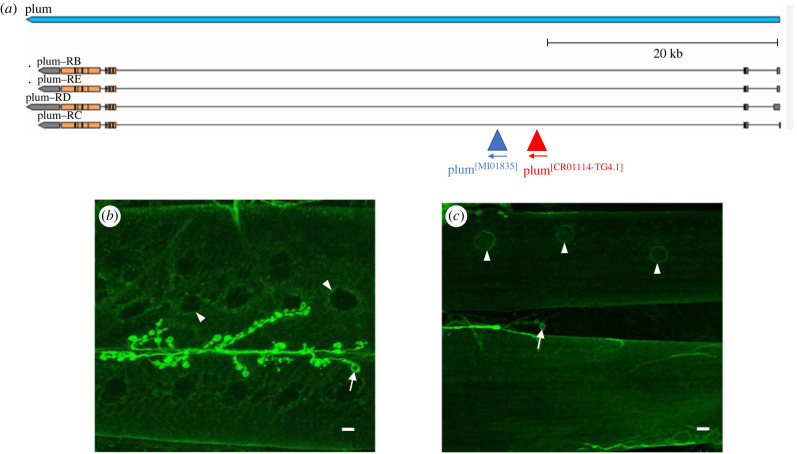
*Plum* is expressed at the postsynaptic NMJ and within muscle. (*a*) *Plum* transcriptional unit (blue) on chromosome 3, showing four isoforms (*Plum-RB,RC, RD* and *RE*), modified from Flybase. Two independent insertion lines were used to detect expression of the *Plum* protein in 3rd Instar larvae. The mimic protein trap line MI01835 (red) and CRIMIC line CR01114-TG4.1 containing the *GAL4* cassette (blue) insertion sites show that these tools detect all *plum* isoforms. (*b*) Protein trap clearly shows *Plum* expression at the neuromuscular junction (arrow) and perinuclear staining in muscle nuclei (arrowhead) as well as low level staining in muscle fibres. (*c*) CRIMIC line driving the expression of membrane localized CD8::GFP shows perinuclear staining (arrowheads) and staining at the NMJ. Note the staining is localized to the outer bouton membrane, indicating postsynaptic localization (arrows in *b* and *c*). Scale bars indicate 10 µm.

We previously showed that genetic attenuation of *Myo* specifically in the larval somatic muscle, using a microRNA construct to target the *Myo* transcript (genotype: *Mef2-GAL4/UAS-miRNAmyo*) [44], increases muscle size, NMJ synaptic transmission and locomotion by greater than 20% [33], defining MYO as a potent neuromuscular inhibitor in flies. Considering the expression of the Plum mammalian homologue Nope in developing skeletal muscles [37] and the microarray data indicating the expression of *Plum* in the body-wall muscles of WT and larvae expressing the human PAX-FKHR protein [45], we first investigated the possible expression of Plum in the larval somatic musculature. Using a previously published rabbit antibody to Plum [36], we visualized the expression of the muscular system of third instar larvae using immunofluorescence, but the result was inconclusive (data not shown). We therefore used a different strategy involving a protein trap (MI01835) that specifically introduced a GFP cassette (flanked with a splice donor and acceptor site) within an intron of the *Plum* transcriptional unit (figure 1*a*). The GFP cassette is spliced into the Plum protein, providing an alternative readout of protein expression. Importantly, the protein would also be expressed at physiologically normal levels as Plum expression is controlled from the endogenous promoter and enhancers. We detected strong expression of the *Plum* gene at the NMJ, particularly around the periphery of the bouton, typical of post-synaptic staining. We also observed weaker expression within muscle tissue (figure 1*b*). The muscle expression of *Plum* was also confirmed using a CRIMIC Gal4 line (CR01114-TG4.1) that also located to the same intron as the GFP protein trap (figure 1*c*). These data showed that the Plum protein could be detected post-synaptically at the NMJ and within the surrounding muscle tissue.

We examined the effect of overexpressing *Myo* and/or *Plum* in muscles. There was no significant electrophysiological differences observed when *Myo*, *Plum* or both were overexpressed in the muscle (electronic supplementary material, figure S1). We then assessed the impact of *Plum* downregulation on NMJ physiology, when expressed in the muscles. We analysed phenotypes in double knock-down *Myo-Plum* larvae (*Mef2-GAL4/UAS-miRNAmyo/plumRNAi*) and single knock-down Plum animals (*Mef2-GAL4/UAS-plumRNAi*).

**Figure 2.**
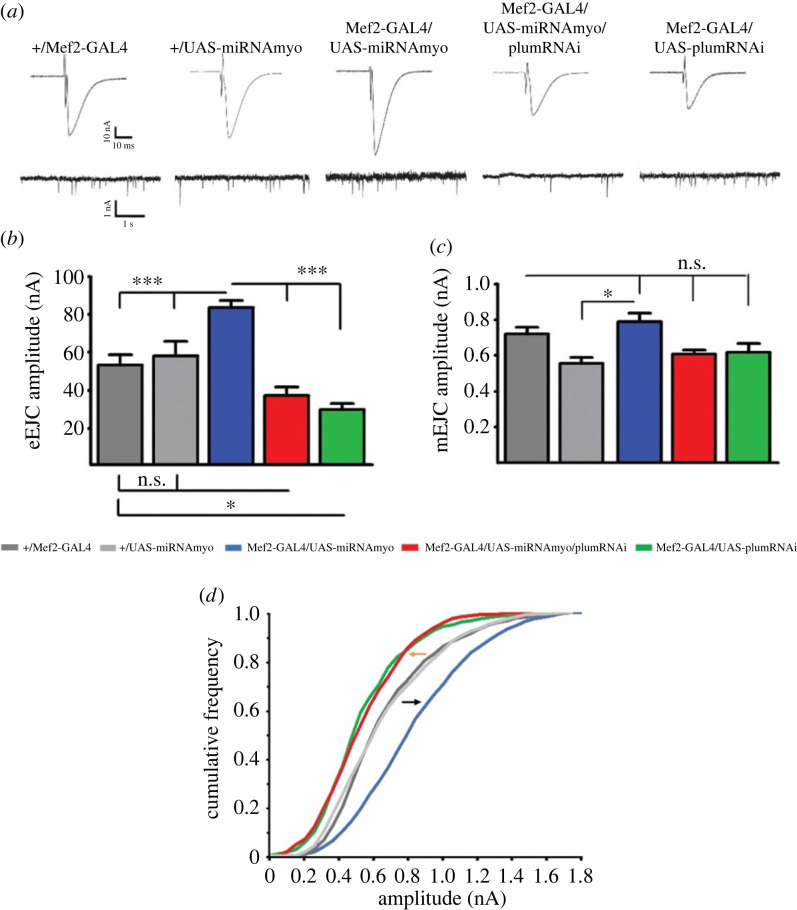
Plum is a regulator of neurotransmission at the larval NMJ. (*a*) Representative traces of evoked (top) and spontaneous (miniature) synaptic responses recorded from muscle 6. Histograms of evoked (*b*) and spontaneous (*c*) responses for given genotypes (control: *+/Mef2-GAL4*; *Myo* downregulation: *Mef2-GAL4/UAS-miRNAmyo*; *Myo* and *Plum* downregulation: *Mef2-GAL4/UAS-miRNAmyo/plumRNAi*; *Plum* downregulation: *Mef2-GAL4/UAS-plumRNAi*) (*n* = 7–16). Two-way ANOVA analysis: the interaction between *Mef2-GAL4/UAS-miRNAmyo and Mef2-GAL4/UAS-miRNAmyo/plumRNAi* is highly significant (*p* < 0.0001). (*d*) Cumulative frequency distribution diagram of mEJC amplitudes. Black arrow indicates shift toward higher amplitude ‘minis’, orange toward lower. For all paired analyses except between *Mef2-GAL4/UAS-miRNAmyo/plumRNAi* and *Mef2-GAL4/UAS-plumRNAi* and between *+/Mef2-GAL4 and +/UAS-miRNAmyo,* KS test, *p* < 0.0001 (*n* = 7–18 animals, ∼1500–2000 events measured per genotype). Bar graphs: error bars indicate SEM (one-way ANOVA + Tukey's post-test: *p < 0.05, ***p < 0.001, n.s. = not significant).

Body-wall muscles in developing larvae consist of bilaterally symmetrical hemi-segments composed of 30 multinucleated muscle fibres [46]. We focused our analyses on muscles 6 and 7, large myofibres innervated by two motoneurons forming a single, excitatory, glutamatergic NMJ. Contractions of these muscles are triggered by ‘non-spiking’ postsynaptic potentials that are graded in duration and amplitude, allowing for quantitative comparisons between genotypes [47]. The amplitude of these Ca^2+^-dependent, nerve-evoked postsynaptic excitatory junctional currents (eEJCs) reflects either the magnitude of presynaptic release or the postsynaptic sensitivity to neurotransmitter [47]. Muscle-specific reduction of MYO leads to dramatically increased evoked response [33]; simultaneous suppression of Plum, however, reversed the response to control (*+ Mef2-GAL4*) levels, with the downregulation of *Plum* alone further diminishing the evoked currents. Further examination of the data showed a statistically significant interaction between the effects of simultaneously reduced MYO and Plum relative to the control genotype (two-way ANOVA analysis in figure 2*b*). We then measured the amplitudes of spontaneous ‘miniature’ postsynaptic currents (mEJCs), also known as ‘quantal size’ [48]. The mEJCs represent postsynaptic responses to a single presynaptically released vesicle containing neurotransmitter and are a reliable indicator of the density of functional, NMJ, glutamate receptors [49]. While the mean mEJC amplitude showed only minor differences between the genotypes (figure 2*a,c*), the frequency distribution analysis revealed that *Plum* downregulation in either control or reduced MYO background caused a strong shift toward smaller ‘miniature’ currents (figure 2*d*). Taken together, our electrophysiological results imply that MYO and Plum affect NMJ physiology by controlling the density of the postsynaptic glutamate receptor field, with Plum having a modulatory effect on MYO and acting as an autonomous synaptic regulator.

### 2.2. Plum modulates the action of MYO on synapse structure and receptor composition

**Figure 3.**
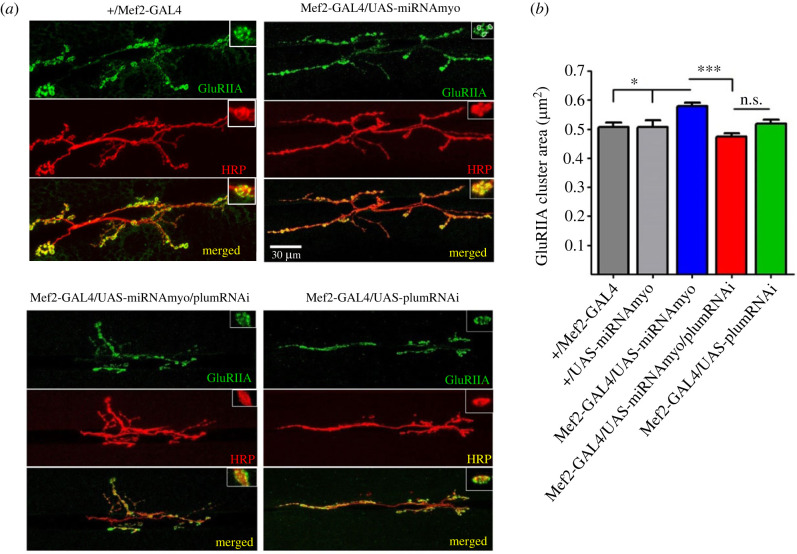
Downregulation of *Plum* reduces the size of GluRIIA clusters in reduced MYO background. (*a*) Representative confocal images of the 3rd instar larval 6/7 NMJs in denoted genotypes. Insets show synaptic varicosities (boutons), with individual GluRIIA clusters within boutons marked by circled areas. Anti-HRP antibody visualized the presynaptic (motoneuronal) membrane. (*b*) Quantification of GluRIIA cluster areas (*n* = 8–9). Bar graph: error bars indicate SEM (one-way ANOVA + Tukey's post-test: **p < 0.01, ***p < 0.001, n.s. = not significant).

Postsynaptic receptors at the larval NMJ are AMPA-type tetrameric complexes formed by glutamate receptor (GluR) subunits IIC, IID and IIE, in addition to either subunit IIA or IIB [50,51]. Assemblies containing the IIA subunit (GluRIIA) are pharmacologically and biophysically distinct from the ones incorporating GluRIIB and carry the bulk of the ionic current at this synapse [48,49]. We have recently shown that elevated evoked and spontaneous synaptic currents in ‘low MYO’ larvae show corresponding increase in the density of GluRIIA receptors [33], in line with previously demonstrated correlation between GluRIIA levels and either evoked response [52] or quantal size [49]. We therefore used immunohistochemistry to measure the area of GluRIIA clusters in the NMJ boutons (figure 3*a*) of control and mutant animals. The GluRIIA cluster area is directly proportional to the number of functional GluRs measured electrophysiologically, and independent of changes in NMJ morphology [53–55].

Our results showed that *Plum* downregulation in the muscle led to significantly (approx. 20%) smaller GluRIIA clusters in animals with muscle-reduced *Myo* expression (figure 3*a,b*). This reduction was identical in magnitude to the increase in the density of IIA-type receptors upon *Myo* downregulation relative to the control genotype [33], demonstrating the reversal to wild-type receptor levels caused by reduced Plum. Unlike its effect on evoked response and distribution of mEJC amplitudes, muscle-specific *Plum* suppression alone was unable to further reduce the GluRIIA cluster area. The lack of perfect correlation between electrophysiological analyses and antibody staining probably occurred because the latter cannot distinguish between functional and non-functional glutamate receptors. Another possible explanation is that, in *Mef2-GAL4/UAS-PlumRNAi* larvae, there is a change in the receptors' biophysical properties resulting in reduced average single-channel conductance [56].

Our immuno-staining data confirm the negative effect of *Plum* downregulation on enhanced neurotransmission caused by muscle-specific knock-down of *Myo*.

### 2.3. The number of Brp puncta scales with NMJ size upon Plum and/or MYO attenuation

Bruchpilot (Brp) is a presynaptic marker at the larval NMJ and the *Drosophila* homologue of the vertebrate active zone protein ELKS [57]. Brp is required for function and structural integrity of synaptic active zones and is necessary for regulating evoked, but not spontaneous, release at the glutamatergic synapse of the NMJ [57].

**Figure 4.**
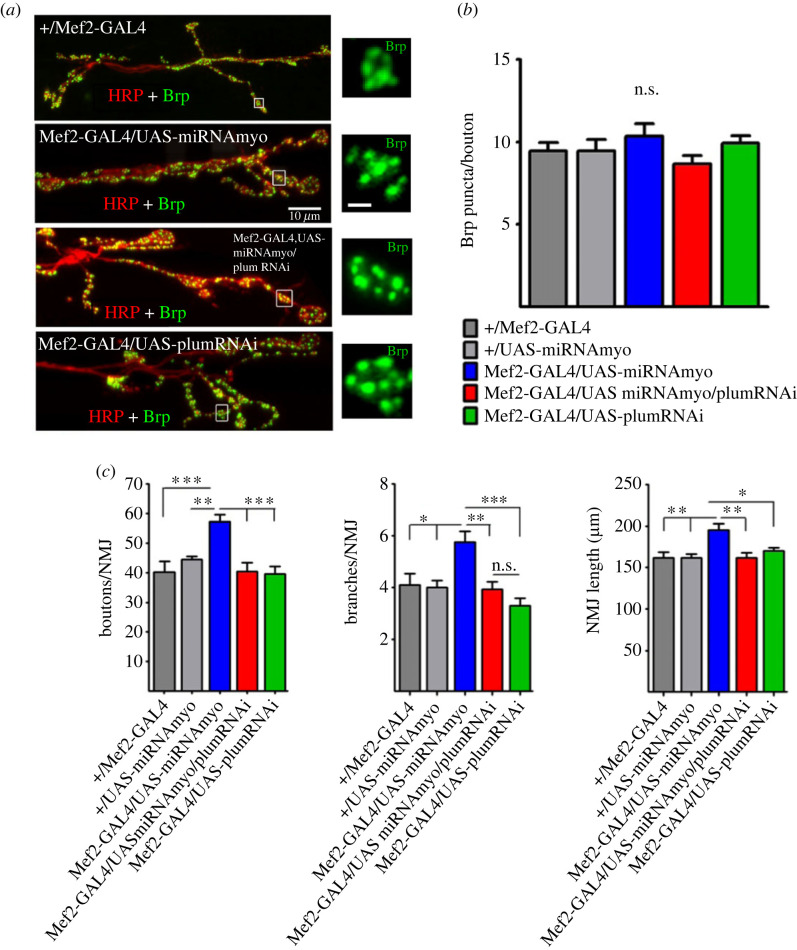
Number of Brp puncta and NMJ morphology. (*a*) Representative confocal images of Brp puncta in distal NMJ segments. Insets show the puncta in individual synaptic boutons (inset scale bar: 1 µm). (*b*) Mean number of Brp puncta per bouton (*n* = 7–9). (*c*) NMJ morphology: the number of boutons (left) and branches (middle) per 6/7 NMJ and NMJ length (right) (*n* = 9–17). All bar graphs: error bars indicate SEM (one-way ANOVA + Tukey's post-test: *p < 0.05, **p < 0.01, ***p < 0.001, n.s. = not significant).

We have previously shown that enhanced evoked response in reduced MYO background correlates with increased NMJ size [33]. These findings agree with the previously established positive correlation between the number of boutons and the strength of evoked signal transmission [52,58]. Here we show that the number of active zones per bouton (active zones marked by distinct Brp puncta) is not affected by *Myo* or *Plum* manipulations (figure 4*a,b*). The reversal of the (increased) amplitude of evoked synaptic responses in reduced MYO larvae upon Plum suppression (figure 2*b*) therefore cannot be explained by reduced number of Brp puncta, despite the previously demonstrated correlation between evoked response and Brp density [59]. Rather, reduced bouton number, NMJ branching, and NMJ length (figure 4*c*) are likely responsible for the physiological attenuation in the larvae suppressing both MYO and Plum. In addition, Plum probably exhibits a broader, non-Brp related, physiological effect because the morphological changes alone cannot explain the negative effect of *Plum* knock-down on synaptic strength in the control background (green bar in figure 2*b*). These findings implicate Plum as a modulator of synaptic strength in low MYO background via its impact on NMJ morphology.

### 2.4. Knock-down of Plum abolishes the effect of reduced MYO on muscle size and body weight

**Figure 5.**
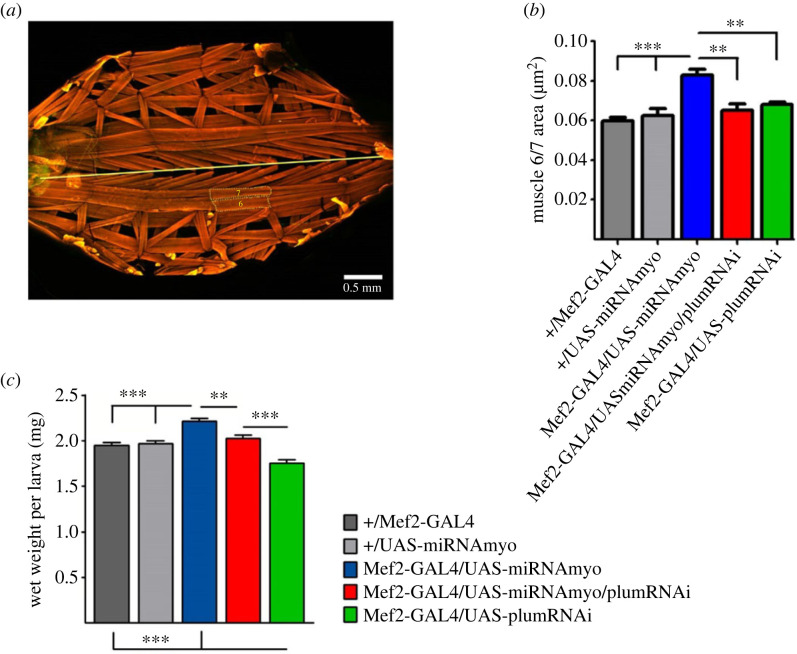
Plum regulates muscle size and larval weight. (*a*) Third instar larval preparation showing body wall muscles stained with phalloidin (anterior is on the left). Straight yellow line marks the midline. Dotted lines mark muscles 6 and 7. (*b*) Bar graphs compare mean combined area of muscles 6 and 7 (*n* = 6–14). (*c*) Total larval wet weight in indicated genotypes (*n* = 19–25). In both (*b*) and (*c*), two-way ANOVA analysis indicates statistically significant interactions between *Mef2-GAL4/UAS-miRNAmyo and Mef2-GAL4/UAS-miRNAmyo/plumRNAi* (*p* = 0.0134 and *p* = 0.0030, respectively). Error bars in the bar graphs indicate SEM (one-way ANOVA + Tukey's post-test: *p < 0.05, **p < 0.01, ***p < 0.001).

Muscle-derived MYO negatively regulates larval weight and muscle size [33]. We examined the effect of Plum attenuation on the size of body-wall muscles 6 and 7 (figure 5*a*) and on total larval body weight in reduced MYO background. Plum suppression completely abolished the positive effect of *Myo* downregulation on the combined area of muscles 6 and 7 (figure 5*b*) and on larval wet weight (figure 5*c*), mirroring its effects on synaptic physiology (figure 2), synaptic composition (figure 3*b*) and NMJ morphology (figures 3*a* and 4*c*). The interaction between *Myo* and *Plum* downregulation was significant, indicating a combinatorial effect of these interventions in abolishing the positive effect of reduced MYO on muscle size and body weight (two-way ANOVA analysis in figure 5*c,d*). Furthermore, Plum has an independent effect on body mass, because *Plum* knock-down larvae exhibit significantly reduced wet weight (figure 5*c*, green bar).

These experimental results identify Plum as a critical modulator of the action of MYO on the neuromuscular physiology, muscle size and weight, and consequently, a regulator of synaptic strength and body weight in developing *D. melanogaster*.

## 3. Discussion

Chemical transmission across the neuromuscular junction is critical for converting action potentials originating in the central nervous system into contractile activity in skeletal muscles. This present work uncovers a previously unknown role for the transmembrane protein Plum in regulating muscle size, muscle weight, GluRIIA receptor clustering and neurotransmission at the larval NMJ when MYO protein is reduced post-synaptically. Previous experiments identified a genetic interaction with MYO and Plum pre-synaptically in the central nervous system, as well as a non-cell autonomous effect from glial cells [36,60]. However, the post-synaptic effects of MYO and Plum had not been addressed.

Post-synaptic overexpression of *Plum* or *Myo* alone in muscle showed no significant effect on the physiology of the synapse (electronic supplementary material, figure S1). The current model of TGF-β signalling suggests that binding of the ligand by type I and Type II transmembrane receptor kinases are required for correct phosphorylation of R-Smad, forming a complex that translocates to the nucleus to initiate transcription of target genes (reviewed by [61]. Wang and colleagues suggest that Plum acts as a coreceptor to stabilize the type I and type II receptors [62]. This would explain why we do not observe any electrophysiological changes when *Plum* is overexpressed in the muscle, as plum is required to stabilize the complex, and there is enough endogenous *Plum* protein to do so. However, Wang *et al.* also show that when they use vCrz neurons as a model to study programmed cell death (PCD), TGF-β signalling is not epistatic to ecdysone signalling, which contrasts with signalling in the mushroom body neurons [36]. Thus, neuron-specific differences exist when addressing TGF-β signalling, although in vCrz neurons, *Plum* does still cooperate with myo to regulate PCD. Further work will be required to understand how *Plum* affects ecdysone signalling in vCrz neurons.

Post-synaptic downregulation of Myo and *Plum*, on the other hand, had a more noticeable effect. *Myo* downregulation resulted in a significant increase in bouton number, branching and NMJ length. *Plum* downregulation ameliorated the effects of *Myo* downregulation and reduced the increases below wild-type levels. Importantly, post-synaptic downregulation of *Myo* increased the number of GluRIIA puncta at the NMJ, which were significantly reduced when *Plum* was concomitantly downregulated. Thus, a homeostatic stabilization of NMJ architecture not only requires MYO and Plum pre-synaptically to ensure normal neuromuscular connectivity before metamorphosis [36], but also involves the interaction of MYO and Plum post-synaptically (this study).

This stabilizing effect is not unique and has also been observed between other secreted proteins and membrane-bound receptors. For example, the disruption of the interaction between the secreted extracellular matrix protein Tenectin and integrin receptor affected NMJ stability both pre-and post-synaptically, and overexpression of Tenectin in muscles not only restored but led to exceedingly high integrin levels [62]. Our data are consistent with the idea that proteins involved with NMJ architecture need to be correctly maintained both pre-synaptically and post-synaptically, and disruption of secreted extracellular molecules may affect NMJ morphology and consequently, NMJ function. Mammalian MYO has been shown to interact with extracellular matrix proteins such as laminin and the extracellular proteoglycan decorin [63,64], suggesting that the regulation of Myostatin by extracellular matrix proteins across the synaptic cleft plays a role in maintaining NMJ stability.

We also showed a significant role for Plum in regulating muscle mass and larval weight, specifically in the context of *Myo* downregulation. Given the role of MYO in regulating NMJ architecture and muscle mass, it is perhaps unsurprising that impairment of the NMJ, in many cases, leads to reduced muscle mass and function in both vertebrates and invertebrates [28,65]. Our work also addressed the consequences of *Myo* and *Plum* downregulation on neurotransmission, using electrophysiological measurements as a readout. Muscle-specific downregulation of *Myo* elicited an increased evoke response, which was attenuated by the concomitant downregulation of *Plum*. These data indicate that tight regulation of Myo and Plum is crucial in maintaining a stable NMJ, and Plum can counteract any potential influence on synaptic strength by deregulated *Myo*, post-synaptically. In addition to motor unit elimination [66], reduced motor axon conduction velocity [67], diminished motor cortex excitability [68,69], and modified activity of muscle-intrinsic factors [70], NMJ dysfunction is strongly correlated with decreased skeletal muscle size and strength under both healthy and pathological conditions [71]. For example, recent studies suggested that the malfunction of the NMJ plays a causative role in the onset of sarcopenia [72], and proposed NMJ stabilization as a way to delay its progression [73]. In dystrophic *mdx* mice, both pre- and post-synaptic abnormalities in the NMJ contribute to reduced muscle contractility [71] and therapeutic approaches that specifically target NMJs have been proposed for treating spinal muscular atrophy and, possibly, ALS [74].

Several mammalian proteins have identified roles in linking structural and functional properties of the NMJ and (skeletal) muscles, the most important being Agrin (no obvious homologue of Agrin is present in *D. melanogaster*). Agrin was identified as a marker for the diagnosis of sarcopenia [75] and implicated in the pathogenesis of sarcopenia caused by degeneration of the NMJ [76]. Degradation of Agrin results in structural changes in the NMJ and innervated muscles, consistent with the notion that impaired NMJ functionality plays a role in the onset of sarcopenia [72]. Agrin was first discovered as a neurotrophic factor sufficient for pre- and post-synaptic NMJ assembly and stabilization [77] and aggregation of the junctional acetylcholine receptors (AChRs) [78]. Agrin binding to transmembrane proteins such as dystroglycan and the extracellular matrix protein laminin is thought to stabilize the NMJ [79,80]. Reduced function and density of synaptic AChRs at the NMJ is a hallmark of ‘normal’ human [12] and rodent [81] ageing muscles and of several NMJ disorders characterized by skeletal muscle loss and weakness [82–84]. These findings underline the importance of investigating links between the NMJ and muscle function and searching for novel regulators of these processes. In the fly, there are several Plum paralogues that show high homology with Plum, and it is tempting to speculate that Plum may form a heterodimeric receptor with one or more of the paralogues to regulate MYO activity. TGF-β ligands are known to bind to different transmembrane receptors, with varying combinations of type I and type II forms [85].

**Figure 6.**
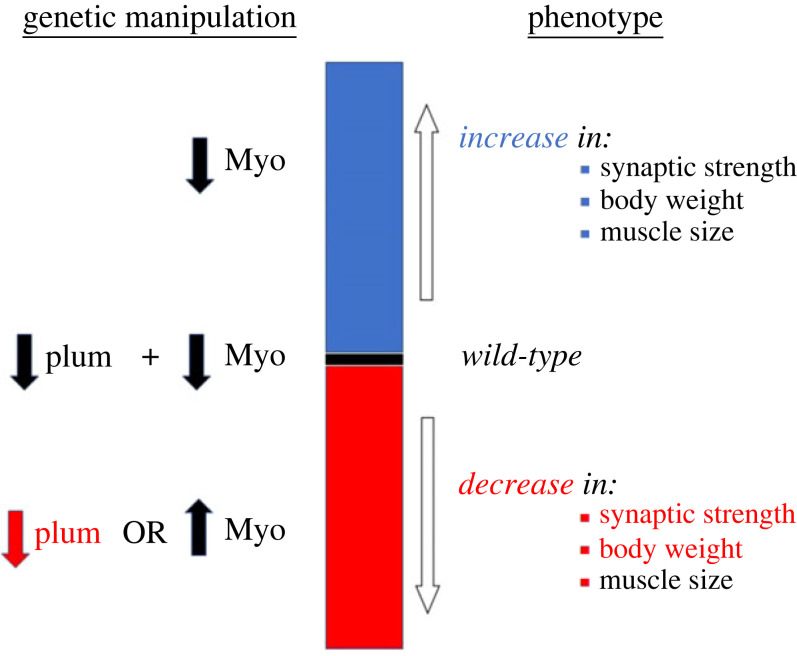
Model illustrating the effects of MYO and Plum on synaptic strength, muscle size and body weight in *Drosophila* larvae. Model showing that Plum reduction abolishes the (positive) effect of reduced MYO on synaptic strength, total body weight and muscle size. Attenuation of Plum on its own, or MYO upregulation, negatively affect synaptic strength and larval weight, with *Myo* overexpression having an additional (negative) effect on muscle size.

Just like Agrin, *Drosophila* MYO is a secreted ligand that regulates nervous system development [44] and NMJ synaptogenesis [33]). While MYO of glial origin governs remodelling of mushroom body neurons in developing animals [44], muscle-derived MYO functions as strong negative regulator of the size of larval somatic muscles and NMJ size and function [33,36]. In this study, we expanded our investigation of the role of MYO in the larval neuromuscular system by analysing its interaction with Plum, a trans-membrane protein recently found to modulate MYO signalling during mushroom body development [36]. The genetic interaction experiments indicate that Plum acts as a downstream effector of MYO possibly by sequestering it and thereby inhibiting its pruning-promoting effect on mushroom body neurons, with the reduction of endogenous Plum stimulating MYO-induced pruning [36]. In the fly brain, *Plum* expression in mushroom body neurons is required to prevent β-lobes from crossing the midline [60]. Overexpression of *Plum* within mushroom body neurons was sufficient to induce β-lobe retraction. However, the overexpression of *Plum* within muscle at the neuromuscular junction was not addressed. We did not see any significant effect of *Plum* overexpression in the muscle when looking at the gross morphology at the NMJ. The only significant effect observed with *Plum* overexpression in the muscle was when *Myo* was also overexpressed. The ‘low MYO’ animals used in our experiments have the *Myo* transcript levels reduced by approximately 60% [33], with the remaining circulating MYO possibly sequestered by Plum. Curiously, work from the O'Connor laboratory [86] did not find MYO functioning as a negative regulator of muscle size. Our work in this paper focuses on the genetic interaction of Plum and MYO, and although we do not directly address the specific function of MYO, we speculate that the differences seen from the O'Connor laboratory data may be due to differences in the genetic background of the flies used. De-sequestering this Plum-bound MYO could therefore reverse some of the effects of genetic MYO attenuation on NMJ function and muscle size. In agreement with this hypothesis, simultaneous suppression of MYO and Plum only in the muscle completely reversed the effect of reduced MYO on synaptic physiology, muscle size, and total body weight. In addition, Plum reduction alone (i.e. without concomitant downregulation of MYO) reduced the synaptic strength and body weight below control levels (figure 6), identifying muscle-derived Plum as a novel and independent target for manipulating neuromuscular function. It is important to note that MYO and Plum regulate physiological properties of the NMJ synapse by controlling the number of glutamate receptors, the functional analogues of ACh receptors in mammalian NMJs. Plum mutant alleles are viable and fertile, indicating that modulating plum levels alone is not detrimental to the development of the fly. Indeed, having little or no phenotypes when plum is deleted or overexpressed is desirable for therapeutic targeting, in order to modify MYO levels.

Sequestration is a well-described mechanism for the regulation of TGF-β ligands, with specific components of the extracellular matrix known to segregate secreted cytokines to either inhibit TGF-β signalling or concentrate the ligands for future use [87]. Sequestration of TGF-β ligands was also demonstrated in *Drosophila* [88] and proposed to facilitate autocrine TGF-β signalling in the larval NMJ [89]. Although the molecular link between MYO and Plum has not yet been elucidated, the model of sequestration currently appears to be the best fit for our observations. Further studies are required to formulate a molecular mechanism and firmly establish this mode of ligand control in regulating the *Drosophila* neuromuscular system. Considering the important role of TGF-β ligands in the regulation of muscle mass and NMJ function in mammalian model species [90,91] and in humans [92,93], these findings can point to novel mechanism for therapeutic interventions in pathologies associated with ageing and neuromuscular disorders.

## 4. Material and methods

### 4.1. Fly stocks and husbandry

All stocks were maintained and all experiments were conducted at 25°C on a 12 h : 12 h light: dark cycle at constant humidity using standard sugar/yeast/agar (SYA) media (15 g l^−1^ agar, 50 g l^−1^ sugar, 100 g l^−1^ autolysed yeast, 100 g l^−1^ nipagin and 3 ml l^−1^ propionic acid) [94]. Third-instar wandering larvae used in the experiments were selected based on morphological (larval spiracles and mouth-hook) and behavioural criteria (location outside of the food). Tissue-specific expression was achieved with the *GAL4-UAS* system [GAL4-dependent upstream activator sequence] [95]. Drosophila stocks used were: *UAS-miRNAmyo* [44], a gift from T. Awasaki from Tzumin Lee laboratory at Janelia Farm; *Mef2-GAL4* (Bloomington Stock Center BDSC #27390); *UAS-PlumRNAi* (Vienna *Drosophila* Resource Centre #101135) and *UAS-Plum^FL^* were a gift from Oren Schuldiner laboratory at Weitzman Institute of Technology. The *Mef2-GAL4/UAS-miRNAmyo/ UAS-PlumRNAi* line was created using standard *Drosophila* crossing schemes. *w^Dah^* was the ‘wild-type’ strain used in all experiments. The white Dahomey (*w^Dah^*) stock was derived by incorporation of the *w^1118^* mutation into the outbred Dahomey background by back-crossing. The plum protein trap line (*plum*^[*MI01835-GFSTF-1*]^) and plum CRIMIC line (*plum^[CR01114-TG4.1^*^]^) were obtained from Bloomington (BDSC stocks #60520 and #81177 respectively). *UAS-IVS-CD8::GFP* was a generous gift from Nic Tapon (BDSC#32185).

### 4.2. NMJ electrophysiology

Recordings were performed as described previously [96]. Two-electrode voltage clamp (TEVC) recordings using sharp electrodes were made from ventral longitudinal muscle 6 in abdominal segments 2–4 of wandering third instar larvae. NMJ recordings were performed using pClamp 10, an Axoclamp 900A amplifier and Digidata 1440A (Molecular Devices, USA) in haemolymph-like 3 (HL-3) solution: 70 mM NaCl, 5 mM KCl, 20 mM MgCl_2_, 10 mM NaHCO3, 115 mM sucrose, 5 mM trehalose, 5 mM HEPES and 2 mM CaCl_2_. Recording electrodes (10–30 M*Ω*) were filled with 3 M KCl. Miniature excitatory junctional currents (mEJCs) were recorded in the presence of 0.5 µM tetrodotoxin (Tocris, UK). All synaptic responses were recorded from muscles with input resistances ≥4 M*Ω* and resting potentials more negative than −60 mV at 25°C as differences in recording temperature cause changes in glutamate receptor kinetics and amplitudes [97]. Holding potentials were −60 mV. Mean single eEJC amplitudes (stimulus: 0.1 ms, 1–5 V) are based on the mean peak eEJC amplitude in response to ten presynaptic stimuli (recorded at 0.2 Hz). Nerve stimulation was performed with an isolated stimulator (DS2A, Digitimer). The data were digitized at 10 kHz and for miniature recordings, 200 s recordings were analysed to obtain mean mEJC amplitudes and frequency values. mEJC and eEJC recordings were off-line low-pass filtered at 500 Hz and 1 kHz, respectively. Materials were purchased from Sigma-Aldrich (UK) unless otherwise stated.

### 4.3. Immunocytochemistry and confocal microscopy

Immunocytochemistry and confocal microscopy were performed as described previously [33] using Zeiss 700 inverted confocal microscope and Olympus FV-1000 inverted confocal microscope. All neuromuscular junction (NMJ) images and analyses were from NMJs on larval ventral longitudinal muscles 6 and 7 (hemi-segments A3-A4). For glutamate receptor (GluRIIA) and Brp staining, 3rd instar larval preparations were dissected in modified HL-3 solution and fixed for 30 min in Bouin's fixative. Mouse monoclonal anti-GluRIIA (8B4D2) and anti-Brp (nc82) antibodies were obtained from the University of Iowa Developmental Studies Hybridoma Bank (Iowa City, USA) and used at 1 : 100 and 1 : 20, respectively. AlexaFluor-conjugated goat anti-mouse secondary antibodies were used at 1 : 200. AlexaFluor-conjugated 488 goat anti-rabbit polyclonal GFP (Cat.no #A21311) was used at 1 : 500 to visualize the plum protein in larval muscles and at the NMJ. TRITC-labelled anti-horseradish peroxidase (HRP) antibody (staining neuronal membranes) was used at 1 : 100. To visualize larval muscles, phalloidin was added to fresh larval preparations fixed for 30 min with 4% paraformaldehyde. Measurements of the postsynaptic glutamate receptors and quantifications of the Brp puncta were made from maximum intensity Z-projections of confocal image stacks using ImageJ (NIH, Bethesda MD). The postsynaptic receptor fields were measured by drawing a circle around individual GluRIIA clusters in type Ib synaptic boutons. NMJ branches, defined as an extension of the presynaptic motor neuron that included at least 3 boutons, were counted manually. NMJ length, defined as the longest end-to-end length across a NMJ, was measured using automated length measurement tool in ImageJ software.

### 4.4. Statistical analyses

Statistical analyses were performed using GraphPad Prism 5 software. For comparisons between two or more groups, a one-way ANOVA followed by a Tukey-Kramer test was used. In all instances, *p* < 0.05 is considered statistically significant (*p < 0.05; **p < 0.01; ***p < 0.001). Values are reported as the mean ± SEM. A 2-way ANOVA test was used to perform interaction calculations. The Kolmogorov–Smirnov (KS) test was used to analyse the cumulative distribution of mEJC amplitudes.

## Data Availability

The data are provided in electronic supplementary material [98].
